# Psychological Effects of Motivational Aquatic Resistance Interval Training and Nutritional Education in Older Women

**DOI:** 10.3390/healthcare9121665

**Published:** 2021-12-01

**Authors:** Alejandro Martínez-Rodríguez, Bernardo José Cuestas-Calero, José Manuel García-De Frutos, Pablo Jorge Marcos-Pardo

**Affiliations:** 1 Department of Analytical Chemistry, Nutrition and Food Sciences, Faculty of Sciences, University of Alicante, 03690 Alicante, Spain; amartinezrodriguez@ua.es; 2 Alicante Institute for Health and Biomedical Research (ISABIAL Foundation), 03010 Alicante, Spain; 3 Physical Activity and Sport Sciences Department, Faculty of Sport, Catholic University San Antonio of Murcia, 30107 Murcia, Spain; bjcuestas@alu.ucam.edu (B.J.C.-C.); jmgarcia887@ucam.edu (J.M.G.-D.F.); 4 Department of Education, Faculty of Education Sciences, University of Almería, 04120 Almería, Spain; 5 CERNEP Research Centre, SPORT Research Group (CTS-1024), University of Almería, 04120 Almería, Spain; 6 Active Aging, Exercise and Health/HEALTHY-AGE Network, Consejo Superior de Deportes (CSD), Ministry of Culture and Sport of Spain, 28040 Madrid, Spain

**Keywords:** exercise, well-being, physical performance, ageing, older adults

## Abstract

Several studies have demonstrated the positive effects of physical exercise programs on physical and mental health throughout life. It is necessary to know the factors that contribute to the well-being of older adults in order to achieve healthy aging. The aim of this study was to evaluate the relationship between well-being perception and the use of autonomy supportive coaching behaviours across a motivational aquatic resistance interval training program. Thirty-four women over 65 years of age from the province of Alicante, Spain, participated, and were randomly assigned to: motivational aquatic resistance interval training group (MART; age: 69.6 ± 5.01 years, height: 1.62 ± 7.88 m, weight: 75.3 ± 12.8 kg) and control group (CG; age: 67.7 ± 3.60 years, height: 1.54 ± 5.47 m, weight: 66.9 ± 10.2 kg). The MART program was conducted for 14 weeks, with three training sessions/week. The CG did not perform any physical activity during the study. Perception of autonomy support was assessed through the Autonomy-Supportive Coaching Questionnaire (ASCQ), Psychological needs by the Basic Psychological Needs in Exercise Scale (BPNES), Intrinsic motivation to exercise was assessed through Intrinsic Motivation Inventory (IMI) and Perception of Physical Activity by the International physical activity questionnaire (IPAQ). In MART, compared to CG, significant differences were observed in BPNS, IMI and IPAQ questionnaires used, except in the ASCQ. The differences were significant in all three cases in BPNS (*p* < 0.05 in autonomy and competence and *p* = 0.001 in relationship with others), obtaining better scores after intervention than previously. As for the IMI scale, significant differences were also obtained in both subscales. The level of physical activity also improved significantly, with higher scores on the IPAQ after the intervention than before (*p* < 0.001). In conclusion, when practitioners perceive greater well-being, satisfaction of their basic psychological needs, greater self-selection, volition, and autonomy instead of pressure, demand and control, the result is better behaviour with greater psychological well-being, adherence and consequent health benefits.

## 1. Introduction

Worldwide, the 65+ age group is growing faster than all younger age groups [1]. To achieve healthy aging, it is important to understand the factors that contribute to the well-being of older adults. “Well-being” is a term that refers to optimal psychological functioning and experience. The Hedonic approach (subjective well-being) focuses on happiness and defines well-being in terms of achievement and avoidance of pain; and the Eudaimonic approach (psychological well-being) focuses on meaning and self-realization, defining well-being in terms of the degree to which a person is fully functioning [2]. 

The Self-determination theory (SDT) [3], is a macro theory of human motivation and personality that concerns people’s inherent growth tendencies and innate psychological needs. This theory focuses on the degree to which an individual’s behavior is self-motivated and self-determined. Being motivated can encourage an individual to do something or persist in a certain behaviour, in a specific context [3]. Where we find two types of motivation: intrinsic motivation would be the inherent tendency to seek novelty and challenge, expand and exercise the ability to explore and learn and which refers to the performance of an activity for the satisfaction inherent in the activity itself. On the other hand, extrinsic motivation refers to the performance of an activity to achieve some result or reward. In addition, some needs are established that are considered psychological and innate (competence, autonomy, and relatedness) and that also motivate the individual to initiate a certain behaviour. Self-determination in older adults has also been defined as a process in which a person has control and ethical/legal rights [4], and as the capacity to make personal choices, irrespective of the person’s ability to accomplish those choices [5].

Basic psychological needs theory (BPN) [2] states that people have three basic psychological needs—autonomy, competence and relatedness—and discusses their relationship to well-being [6,7]. Autonomy defined as the capacity to act and decide in accordance with one’s own free wishes [8]. Psychological health depends on satisfying the three needs [9]. All the needs contribute to effective functioning and to psychological health. None of them could be disclaimed.

Autonomy support is a component of the motivational climate in physical activity that can promote the internalization of behaviours and attitudes by practitioners [10]. In several investigations, autonomy support by the trainer has been related to intrinsic motivation, to more self-determined extrinsic motivations and to the intention to continue practicing a physical exercise program [11,12]. Other studies [13,14] consider the satisfaction of basic psychological needs as a mediator of practitioners’ well-being. 

Indeed, physical activity is considered one of the main determinants of successful ageing, and an abundance of research on physical activity suggests that when people are autonomously motivated to exercise, they are most likely to do so [4,15,16,17,18,19]. So, when older adults engaging in physical activity are more able to satisfy all their needs, the regulation of their behaviour is characterized by choice, volition, and autonomy rather than pressure, demand, and control, and the result is higher quality behaviour and greater psychological well-being [20,21]. In the older adults, resistance training should be considered as a very effective preventive strategy to delay and attenuate the negative effects of sarcopenia and frailty, both in the early and late stages [22]. As presented in this Position Statement [23], current research has demonstrated that countering muscle disuse through resistance training is a powerful intervention to combat the loss of muscle strength and muscle mass, physiological vulnerability, and their debilitating consequences on physical functioning, mobility, independence, chronic disease management, psychological well-being, quality of life, and healthy life expectancy. Furthermore, Felipe Garcia-Pinillos et al. [24] demonstrated that interval-based endurance training (HIT) leads to greater improvements in body composition, muscle strength, mobility, balance and thus quality of life, perception, and autonomy, in healthy older people than regular low-moderate intensity continuous training, despite the reduction in overall training volume.

In addition, there is strong evidence to support the use of aquatic exercise to achieve physical benefits such as improved aerobic capacity, strength, psychological and social benefits in older adults [25,26,27,28]. Exercise in aquatic environment has a lower risk of traumatic fracture, lower joint impact, lower load due to buoyancy, compared to land-based exercise [29]. This type of sport has been recommended for the reduction of pain and greater safety in patients with disabilities. Several studies show that if the teacher uses motivational strategies that foster self-determined motivation, the trainees achieve greater interest and adherence to the program [30,31,32,33].

Therefore, the purpose of the current study was to evaluate the relationship between the well-being and the use of autonomy supportive coaching behaviours across a motivational aquatic resistance interval training program. A secondary purpose was to investigate differences in perceived autonomy support, basic need satisfaction, and intrinsic motivation among older women. The initial hypothesis is that upon exposure to trainings on autonomy supportive coaching, participants’ levels of autonomy support, relatedness support, competence and well-being would improve, while controlling behaviours and relatedness thwarting would decrease. With regards to the practitioners, it was expected that changes in perceptions of autonomy support, satisfaction of basic needs, and intrinsic motivation would improve across the program.

## 2. Materials and Methods

### 2.1. Design

This study is a randomized clinical trial consisting of two groups: experimental group: motivational aquatic resistance interval training group (MART) and control group. Following published recommendations [34] subjects were randomized electronically by block design into two arms (MART group, and control group) using online computer software. A researcher who was not involved in the assessments or interventions of this study prepared this sample distribution. 

### 2.2. Participants

**Figure 1:**
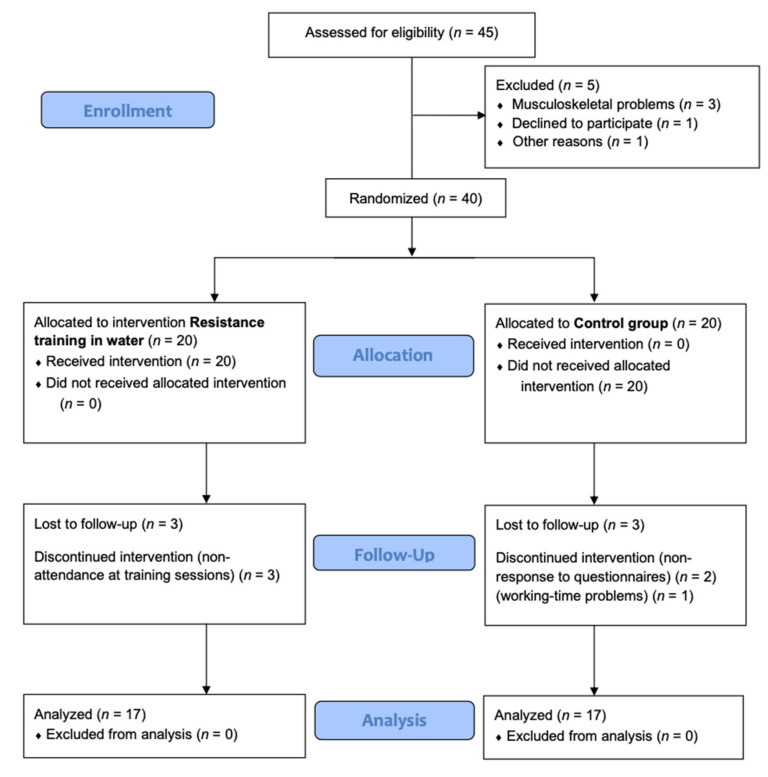
Consort 2010 flow diagram.

Thirty-four women over 65 years of age (67 ± 4.31 years) from the province of Alicante, Spain, participated in the study. The inclusion criteria were as follows: (a) female participants; (b) over 65 years of age; (c) non-institutionalized older women; (d) who had the autonomy to travel from their residence to the municipal swimming pool; (e) individuals who gave their permission to participate in the study by signing the consent form; (f) individuals with medical authorization for the practice of physical exercise in an aquatic environment. Exclusion criteria were (a) being under 65 years of age; (b) having completed a physical exercise program; (c) suffering from musculoskeletal, neurological, or orthopaedic diseases that could affect the ability to perform the tests; (d) not being able to walk independently without orthopaedic assistance; (e) having previously performed the tests included in the study; and (f) be receiving a pharmacological treatment that may influence the results of the investigation. Over the follow-up period, six participants withdrew from the trial. Seventeen women participated in the experimental group and seventeen in the control group. All withdrawals were due to personal reasons (Figure 1).

This research was conducted according to the standards of the Helsinki Declaration and was approved by the University Human Research Ethics Committee of Catholic University of Murcia (Spain) code CE061920. The study protocol was registered (retrospective registration) in the Australian New Zealand Clinical Trials Register database (https://www.anzctr.org.au/ (accessed on 26 November 2021)) with the request number: 383,201 (date of registration: 26 November 2021). All participants signed an informed consent form after being informed of the benefits, risk, and detailed description of this research. All data were coded to maintain the confidentiality of the study participants. 

### 2.3. Intervention

**Figure 2:**
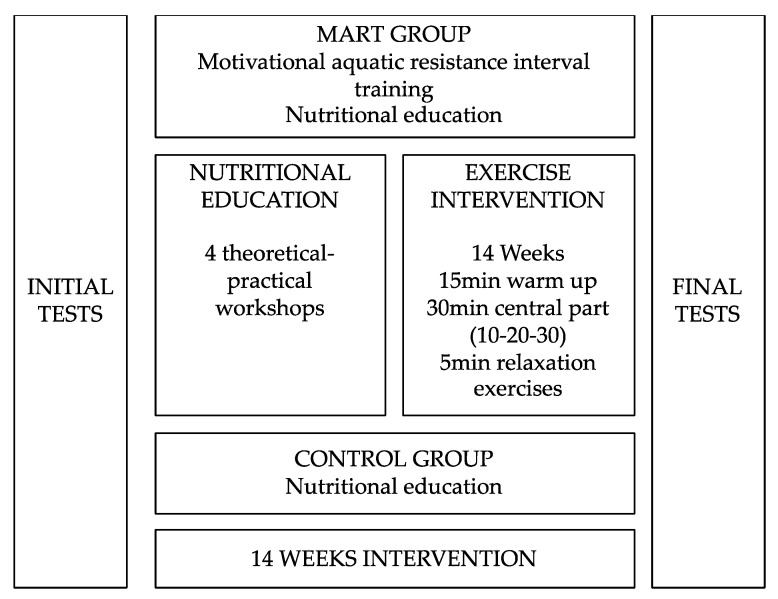
Summary of intervention.

Prior to the intervention, a first session was held where the participants were given different tests (pre-test). After the intervention, another evaluation was carried out to determine the effect of the intervention (post-test) (Figure 2). The MART group performed an aquatic resistance and motivational training, and the control group did not perform any type of exercise. The water temperature was approximately 29 °C (84 °F), and the minimum water depth was considered 1.3 m.

The training intervention consisted of a 14-week of aquatic resistance training program developed using motivational strategies. Each session had a duration of 60 min and was performed 3 times a week (48 h between each) in a heated pool. The first 15 min of the session consisted of aerobic endurance exercises (10 min) and stretching (5 min) for all muscle groups involved. Then, the central part of the training (30 min) consisted of an integral interval resistance training in an aquatic environment. This intervallic training consisted of 4 sets of 5 min of training with 2 min rest between sets. In each set, the same exercise (pectoral/back, hip flexor/extensor, biceps/triceps, knee flexor/extensor, shoulder and core) was performed for 1 consecutive minute with intervals divided into 30, 20 and 10 s performed at maximum intensity [35], and at low, moderate and high perceived intensity respectively [36]. According to the perceived exertion scale, when participants needed to increase the intensity of the upper hemisphere exercises, they put on resistance gloves or resistance dumbbells, whereas for the lower hemisphere exercises they put on resistance anklets.

Finally, relaxation exercises (10 min) and stretching of all muscle groups (5 min) were performed. In addition, the whole process was supervised by a Physical-Sports Educator and in all intervention sessions the perception of effort was monitored using the Borg scale [37]. During the training program sessions, the teacher used motivational strategies as previously published, designed to increase the motivation of the older adults towards the aquatic resistance training program [30,33,38]. In order to make the aquatic resistance training program motivating and increase adherence, a series of motivational strategies from the previously cited studies were adapted and implemented. 

Strategies based on autonomy:

1. Educate older adults about the benefit of aquatic resistance training program;
2. Explain the purpose of the aquatic resistance training program;

Strategies based on competence;

3. Encourage the perception of competence by the participant;
4. Establish moderately difficult objectives adapted to the biological individuality of each participant;
5. Take into account the information provided by the practitioner during aquatic resistance training program;
6. Convey an adequate task environment;
7. Encourage the participants by emphasizing that the activity can be improved through practice;
8. Offer clear feedbacks;

Strategies based on social relations and enjoyment;

9. Encourage the relationship between participants;
10. Have the participants enjoy the activities in aquatic resistance training program.

In addition, all participants received nutritional education, without any difference. The nutritional intervention was based on four theoretical and practical workshops of 60 min for 14 weeks, whose purpose was to provide updated information about the benefits of following an adequate food pattern. 

In addition, all participants received the same nutritional education, based on the Mediterranean diet (MD), to provide updated information on the benefits of following an adequate dietary pattern. Four 60-min theoretical-practical workshops were held over the 8 first weeks. Trained dietitians led the sessions. Several topics were covered in the sessions. Workshop 1; nutrition and food, DM pyramid, daily, weekly, and occasional dietary guidelines to achieve a healthy and balanced diet adapted to different daily situations. Workshop 2; health and gastronomy-preparation of healthy menus including DM components with impact on cardiovascular prevention and cognitive impairment. Workshop 3; healthy aging and Mediterranean diet, hydration, macronutrients and foods, micronutrients. Workshop 4; different types of sugars, effects of sugar consumption, processed products, risk of associated cardiovascular diseases, reading labelling. All participants attended all sessions, thus avoiding those dietary habits were a potential confounding factor of the results obtained as an effect of the training.

### 2.4. Outcomes

#### 2.4.1. Perception of Autonomy Support

The Autonomy-Supportive Coaching Questionnaire (ASCQ) [10] was used to measure the perception of autonomy support. This questionnaire consists of nine items and assesses two forms of autonomy support: interest in practitioners’ contributions and praise for autonomous behaviour. Responses to these items are made on a 7-point Likert scale, with 1 (not at all true) and 7 (very true). Higher scores indicate greater autonomy. Internal consistency analysis was performed using Cronbach’s alpha, 0.908 for the interest in athlete’s opinion scale and 0.902 for assessment of autonomous behaviour. 

#### 2.4.2. Psychological Needs

The Basic Psychological Needs in Exercise Scale (BPNES) [39] in its Spanish version [40] was used to assess psychological needs. This questionnaire consists of 12 items with three subscales: autonomy, competence, and relatedness. The score of this questionnaire was obtained from the response to the items through a Likert-type scale ranging from 1 (strongly disagree) to 5 (strongly agree). Higher scores mean higher levels of autonomy, competence, and relationships with others. Reliability of the questionnaire subscales showed Cronbach’s α = 0.953 for the autonomy, α = 0.955 for competence, and α = 0.955 for relationship with others. 

#### 2.4.3. Intrinsic Motivation to Exercise

Intrinsic Motivation Inventory (IMI) [41,42] is a multidimensional scale that assesses motivational structures for specific activities (sports, school, laboratory tasks, etc.). It is composed of 45 items divided into 6 subscales: “interest/enjoyment”, “perceived competence”, “effort/importance”, “pressure/tension”, “perceived choice”, “value/usefulness” and “relatedness”. The questionnaire is scored on a 7-point Likert-type scale, with 1 (strongly disagree) and 7 (strongly agree). The scores for each subscale are obtained by averaging the different items. Higher scores mean higher motivation. Internal consistency at IMI questionnaire presented the Cronbach’s alpha coefficients 0.931 and 0.903 for enjoyment and effort subscales, respectively.

#### 2.4.4. Perception of Physical Activity

The International physical activity questionnaire (IPAQ) [43,44] long version is a 27-item questionnaire used to assess subjects’ physical activity and includes a wide range of physical activities. All activities performed during the previous 7 days are included: occupational physical activity; transport physical activity; household chores, housekeeping and family care; recreational, sport and leisure physical activity; and time spent sitting or not being active [43], such that higher scores indicate a higher level of activity.

#### 2.4.5. Anthropometric Measurements

The weight and height of all participants were measured using high-quality electronic calibrated scales and a wall-mounted stadiometer, respectively. Both measurements were determined with participants wearing light clothing and no shoes.

### 2.5. Statistical Analysis

Jamovi 1.1.3.0 software was used to perform all statistical analyses. Descriptive statistics (mean ± standard deviation) were calculated for all variables and the normality distribution was tested using the Shapiro–Wilk test. Independent samples t-tests were performed to compare baseline values between groups. Subsequently, Levene’s test was performed for equality of variances and analysis of covariance (ANCOVA) (general linear model; time × group) with body mass index (BMI) as a covariate was applied to analyse the effects of the intervention on the assessments. For time × group interaction effects, partial eta squared effect sizes (η^2^) were calculated (η^2^ ≥ 0.01, indicates a small effect, ≥0.059 a medium effect and ≥0.138 a high effect). If significant main effects were found, post hoc (Bonferroni) tests were performed. The level of statistical significance was set at *p* ≤ 0.05. Finally, the effect size (ES) was calculated following Cohen’s guidelines [45]. The ES was interpreted as small if it obtained values between 0.2–0.5, moderate if 0.5–0.8 and large when >0.8. 

## 3. Results

Table 1 contains information about the characteristics of the sample. Statistically significant differences are observed between the MART group and the control group in height and weight, being higher in both cases in the resistance training group. For this reason, BMI has been considered as a covariate in the statistical analysis.

Table 2 presents the summary statistics of the ANCOVA analysis. The main analysis of the present study shows that there was a significant time × group difference (*p* < 0.001) in all the variables analysed. In all cases, the results are better in the MART group.

**Figure 3:**
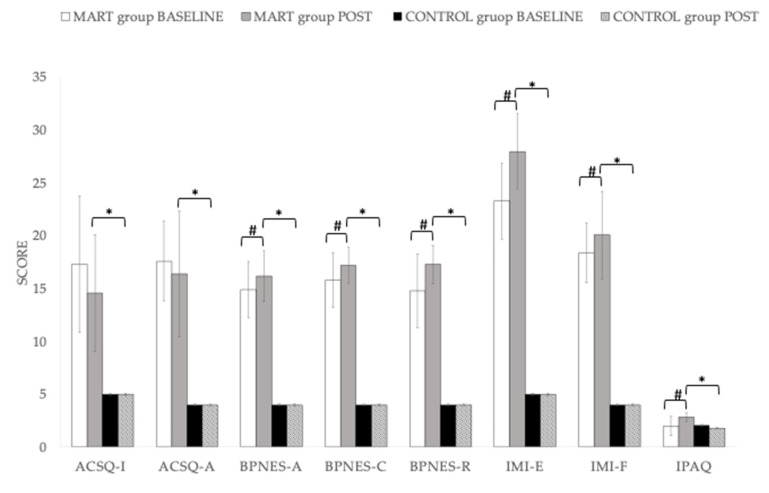
Sample characteristics at baseline and post-intervention. ACSQ-I = ACSQ Interest in the athlete’s opinion; ACSQ-A = Assessment of autonomous behavior; BPNES-A = Basic Psychological Needs in Exercise Scale—Autonomy; BPNES-C = Basic Psychological Needs in Exercise Scale—Competence; BPNES-R = Basic Psychological Needs in Exercise Scale—Relationship with others; IMI-E = Intrinsic Motivation Inventory—Enjoyment; IMI-F = Intrinsic Motivation Inventory—Effort; IPAQ = International Physical Activity Questionnaire; * = Significant difference between MART group vs. control group; # = Significant difference baseline vs. final. Differences were significant at *p* < 0.005.

In the case of the control group, there were no differences between pre and post in any of the variables studied, as shown in Figure 3 and Table 2. However, in the intervention group, significant differences were observed in most of the questionnaires used, except in the ASCQ. In neither of the two scales, “Interest in the athlete’s opinion” and “Assessment of autonomous behaviour” are significant differences in the experimental group. In the control group the minimum scores were obtained. 

In the Basic Psychological Needs Measurement Scale (BPNES), three subscales were analysed: autonomy, competence, and relationship with others. The differences were significant in all three cases (*p* < 0.05) in autonomy and competence and *p* = 0.001 in relationship with others), obtaining better scores after the intervention than previously. As for the IMI scale, significant differences were also obtained in both subscales. The level of physical activity also improved significantly, with higher scores on the IPAQ after the intervention than before (*p* < 0.001).

## 4. Discussion

The main purpose of the present study was to assess the relationship between the well-being perception and the use of autonomy supportive coaching behaviours across a motivational aquatic resistance interval training program. As well as a secondary purpose was to investigate differences in perceived autonomy support, basic need satisfaction, intrinsic motivation, and physical activity among older women.

After the present study, it appears that a MART program developed by a professional with a degree in Physical Activity and Sport Sciences (Physical-Sports Educator) has benefits in the psychological variables studied. As stated by Margie E Lachman et al. [33] cognitive-behavioural and motivational approaches are needed to increase the likelihood of lasting behaviour change by increasing motivation, self-efficacy, and sense of control over physical activity, using a personalized approach, with social support and meaningful goal setting. The positive effects of physical activity on the physical and psychological well-being and life satisfaction of older adults are evident [1,46,47,48,49,50]. However, the novelty of this project was to integrate motivational strategies previously published within the intervention of physical exercise training in water in older women. It should be noted that the ASCQ score decreased from 17.3 ± 6.43 to 14.6 ± 5.51 for the “Interest in the athlete’s opinion” scale and from 17.6 ± 3.78 to 16.4 ± 5.95 for the “Assessment of autonomous behavior” scale. This was to be expected, given that since the classes were programmed and directed, it was not the participants who chose what and how to do the exercises.

SDT [3] suggests that when participants psychological needs for autonomy, competence, and relatedness are meet in a physical activity context, they will experience more self-determined types of motivation. With ageing and, in consistent with SDT, show that autonomy and relatedness need satisfaction is positively associated with indicators of well-being such as purpose in life and personal growth, considered as essential components of optimal functioning [51]. In the present study, participants who performed resistance training showed significant (*p* < 0.001) results of increased autonomy (14.9 ± 2.67 and 16.2 ± 2.41 points, before and after, respectively) competence (15.8 ± 2.58 and 17.2 ± 1.73 points) and social relationships (14.8 ± 3.49 and 17.3 ± 1.81 points), as well as an increase in intrinsic motivation after the intervention. This confirms the conclusions reached by Conroy, D.E et al. [10] that autonomy support is a component of the motivational climate in physical activity that can promote the internalization of behaviours and attitudes on the part of participants. 

In relation to the present work, a previous study found that the perception of psychological well-being and the improvement of subjective health were also significantly related to regular and intensive physical exercise [52], another recent study indicates that daily physical activity has psycho-social benefits in the older adults and may lead to better outcomes in the primary prevention of disability [53]. The improvements reported by the experimental group coincide with those of other aquatic training programs developed in older adults, who also obtained improvements in functional autonomy [54], although in their case, they did not carry out psychological strategies. These strategies may also have contributed to the fact that the study sample completed all the sessions, except for 3 people who, for personal reasons, had to be excluded from the study. 

This may be due to an improvement in adherence to sports practice, which is achieved by incorporating motivational strategies into physical exercise training [55]. In addition, some authors proposed alternative forms of exercise to increase the adherence to physical exercise programs, as our aquatic resistance training program [56], and not just to improve the autonomy and competence, but the relationship with others as well. 

In addition, although the effort was quite high in some parts of the training session, the intensity session average was moderate, and that fact, may help as well to have a good adherence to the program, and could succeed and achieve good scores of well-being. Participants also reported an intrinsic motivation associated with greater well-being. In fact, a significant improvement (*p* < 0.001) is observed both in the “enjoyment” scale, increasing the score from 23.3 ± 3.61 to 28.0 ± 3.58 and in the “effort” scale from 18.4 ± 2.81 to 20.1 ± 4.15 points on the IMI questionnaire, which measures intrinsic motivation.

Another recent study indicates the potential of moderate physical activity in promoting emotional well-being and highlights the role of exercise as cost-effective opportunities for older adults to socialize and improve their emotional functioning [21]. 

Some limitations of the present study need to be addressed. First, the small sample size used, which also included only physically active female volunteers, mean the results may not be generalizable to all older adults. Second, the intervention focused on older adults participating in a specific aquatic resistance training program. Future research should determine whether the results are the same in other physical activity programs. Third, it should be measured whether more education can indirectly increase awareness of health benefits and increase the importance of engaging in physical activity among middle- and upper-class individuals. Another limitation is that we did not create a third training group that did not receive the motivational strategies because we preferred that the larger sample benefit from this program. In this sense, the results cannot be extended to a demographically broader older adult. Fifth, nutritional education intervention was not evaluated at the end of the lessons, so we are unable to evaluate the real effect of this intervention on the subjects’ habits and the last limitation, the study was not registered prospectively. Despite these limitations, this study provides important support for understanding self-determined behaviours and the relationship between need satisfaction and well-being in the aquatic resistance training program.

## 5. Conclusions

Results from the present study provided evidence for the beneficial effects of MART program in older adults. Thus, when physically active older adults are more able to perceive greater well-being and meet their basic psychological needs, their behavioural regulation is characterized by self-choice, volition, and autonomy rather than pressure, demand, and control, and the result is higher quality behaviour and greater psychological well-being. Further research is needed to extend these findings and to better understand the motivational processes of older adults in aquatic and land-based physical exercise programs to help them maintain an active lifestyle and achieve healthy aging.

## Figures and Tables

**Table 1:** Baseline characteristics of study participants.

	MART Group (*n* = 17)			Control Group (*n* = 17)			Baseline Differences	
	Baseline			Baseline				
	Mean		SD	Mean		SD	t	*p*
Age (Years)	69.6	±	5.01	67.7	±	3.60	1.257	0.218
Height (cm)	162	±	7.88	154	±	5.47	3.347	0.002 *
Weight (kg)	75.3	±	12.8	66.9	±	10.2	2.122	0.042 *

MART = aquatic resistance and motivational training; SD = standard deviation; * Mean differences were significant at *p* < 0.05.

**Table 2:** Comparison characteristics at baseline and post-intervention (ANCOVA).

	Effect Time				Effect Time × Group			
	MD	SE	*t*	*p*	MD	SE	*t*	*p*
ACSQ Interest in the athlete’s opinion	1.43	0.75	1.90	0.066	9.36	1.38	6.76	< 0.001
ACSQ Assessment of autonomous behaviour	0.63	0.74	0.85	0.403	12.26	1.22	60.4	< 0.001
BPNES Autonomy	−0.63	0.34	−1.87	0.070	12.15	0.62	19.55	< 0.001
BPNES Competence	−0.74	0.33	−2.24	0.032	13.22	0.53	24.89	< 0.001
BPNES Relationship with others	−1.29	0.47	−2.73	0.010	13.31	0.69	19.39	< 0.001
IMI Enjoyment	−2.40	0.69	−3.47	0.001	23.15	0.86	26.96	< 0.001
IMI Effort	−0.83	0.46	−1.80	0.080	16.32	0.80	20.29	< 0.001
IPAQ	−0.31	0.15	−2.05	0.049	1.01	0.25	50.70	0.001

ACSQ = Autonomy-Supportive Coaching Questionnaire; BPNES = Basic Psychological Needs in Exercise Scale; IMI = Intrinsic Motivation Inventory; IPAQ = International physical activity questionnaire. Mean differences were significant at *p* < 0.05; SE = effect size; *t* = *t* student.

## Data Availability

The data presented in this study is available on request from the cor-responding author. The data are not publicly available due to is personal health information.
